# Supplementary material for: The Presence of Blood–Brain Barrier Modulates the Response to Magnesium Salts in Human Brain Organoids
Source: Int J Mol Sci. 2022 May 4;23(9):5133. doi: 10.3390/ijms23095133 (PMC9104490; doi:10.3390/ijms23095133)
Supplement: Supplementary file 1 [file ijms-23-05133-s001.zip › ijms-1682297-supplementary.pdf]

The presence of BBB modulates the response to magnesium salts in human brain organoids

Alessandra Cazzaniga, Giorgia Fedele, Sara Castiglioni and Jeanette A. Maier

Supplementary

1 and 5 Mm of pidolic acid were tested on brain organoids cultured under BBB as control. The results obtained by Real Time PCR show that the pidolic acid does not exert any effect on the expression of *NMDA-R*, *GABA-Rs* and *BDNF*, thus highlighting the important role of the MgPid in the observed modulations.

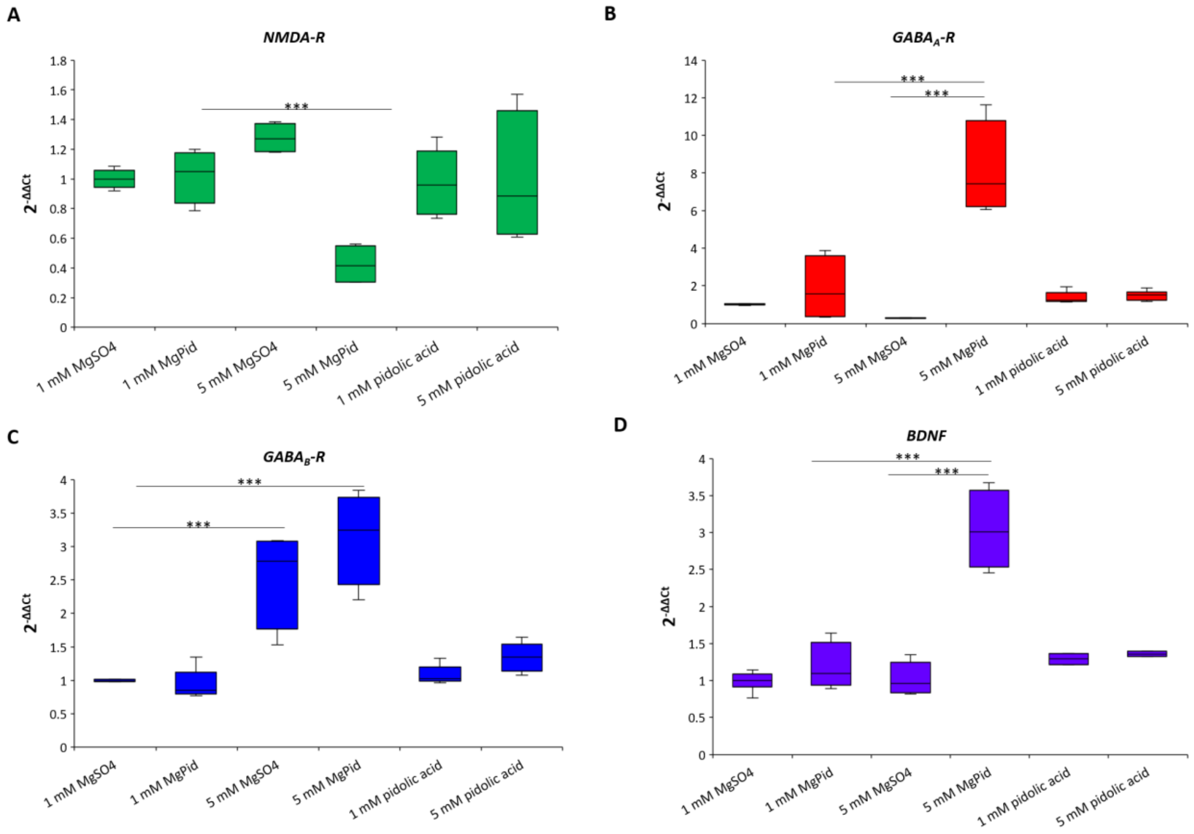

Figure S1. Brain organoids treated with pidolic acid. Human brain organoids were cultured with or without the BBB, and exposed to 1 or 5 mM MgPid, $MgSO_4$ or pidolic acid for 4 days. Box plot shows the Real Time PCR performed three times in triplicate on RNA extracted from the brain organoids. Each experimental sample was obtained by pooling three organoids. Primers were designed on (**A**) *GRIN1* (*NMDA-R*), (**B**) *GABRA2* (*GABA_A-R*), (**C**) GABBR1 (*GABA_B-R*) and (**D**) *BDNF* sequence. The mRNA expression values were normalized to *GAPDH* mRNA levels. All the experiments were performed at least three times in triplicates. * $p \leq 0.05$, ** $p \leq 0.01$, *** $p \leq 0.001$.
